# Ca-HELP: Adaptation of a Communication Tool to Help Geriatric Cancer Patients in Rural Settings Talk to Their Doctors About Pain

**DOI:** 10.1093/geroni/igad087

**Published:** 2023-08-22

**Authors:** Jill Harrison, Tammy Stokes, Jessica Hahne, Megan Shen

**Affiliations:** Department of Health Services, Policy, and Practice, School of Public Health, Brown University, Providence, Rhode Island, USA; Maury Regional Medical Center, Columbia, Tennessee, USA; Department of Psychological & Brain Sciences, Washington University in St. Louis, St. Louis, Missouri, USA; Clinical Research Division, Fred Hutchinson Cancer Center, Seattle, Washington, USA

**Keywords:** Adaptation, Cancer, Older adults, Pain, Stakeholders

## Abstract

**Background and Objectives:**

Among the older adult population living in the rural United States, undertreated cancer pain is very common. The need for interventions targeting pain management communication between older adults with cancer in rural communities and their doctors outpaces the current evidence base. Adaptation of existing pain interventions may improve the speed at which clinicians can respond to pain in this vulnerable population.

**Research Design and Methods:**

The Cancer Health Empowerment for Living without Pain (Ca-HELP) is an evidence-based communication tool that coaches patients to communicate about pain by asking questions, making requests, and signaling distress to their physicians in order to achieve improved pain control. Guided by the Method for Program Adaptation through Community Engagement (M-PACE) model, which utilizes detailed stakeholder feedback to guide the adaptation of an intervention for an appropriate target audience, we proactively adapted the Ca-HELP and its delivery for use among geriatric cancer patients living in rural settings using qualitative feedback from patients, informal caregivers, and providers as a planned step in a multiphase pilot study.

**Results:**

All stakeholders agreed that the Ca-HELP was a promising candidate intervention to improve pain among older adults with cancer. They suggested modifications to the delivery, context, and content of the intervention. A multidisciplinary team of nurse leaders and researchers evaluated stakeholder feedback and recommendations before determining which adaptations were made. Adaptations were cataloged and reported using the Framework for Reporting Adaptations and Modifications-Enhanced model.

**Discussion and Implications:**

Our multistakeholder team proactively modified the Ca-HELP intervention tool using end-user feedback with a goal to optimize fit for use by older adults with cancer in rural settings without compromising the active ingredients. Documenting and reporting modifications to interventions are critical to their implementation and will lay the groundwork for further testing of the efficacy of the adapted Ca-HELP intervention.

**Translational Significance:** Among the growing older adult population living in the rural United States, undertreated cancer pain is very common and patients are hesitant to ask for pain relief. There is an urgent need for pain management interventions that can be implemented effectively among these vulnerable communities. Adapting existing evidence-based interventions for these populations may improve the speed at which this need can be addressed. Modifying a candidate intervention is a social enterprise that involves planning and partnership between researchers and stakeholders. Documenting and reporting modifications to interventions are critical to preserving their active ingredients and optimizing their fit and implementation.

## Background and Objectives

Effective pain management is one of the largest population health challenges among older adults in the United States (Gloth, 2001; Nawai et al., 2017; Park et al., 2015, 2016; Reid et al., 2008; Robinson-Lane & Vallerand, 2018; Schofield, 2017; St Marie & Arnstein, 2016). Older adults living in rural areas are disproportionately affected due to the following factors: concerns about the over-use of pain medication (Nawai et al., 2017), less access to care (Park et al., 2016), patients’ preferences for nonpharmacological interventions (Robinson-Lane & Vallerand, 2018), and patients’ reluctance to talk about pain (Cagle & Bunting, 2017). Among the older adult population living in the rural United States, undertreated cancer pain is very common (Reyes-Gibby et al., 2006). Older patients often hide symptoms of pain and avoid communicating their need for pain relief (Beck et al., 2009; Cleeland, 1998; Dalton et al., 1995; Hachem et al., 2019; Reyes-Gibby et al., 2006). Older adults with cancer are commonly afraid to voice pain concerns as they believe it reflects worsening cancer, is just part of life, and do not want to burden others (Hachem et al., 2019). Pain management communication between patients and doctors in rural settings exists against the backdrop of a growing opioid crisis disproportionately affecting these communities (Harbaugh et al., 2017; Keyes et al., 2014). There is an urgent need for interventions targeting pain management communication between older adults with cancer in rural communities and their doctors; however, this need outpaces the current evidence base targeting this vulnerable population. Adaptation of an existing evidence-based pain intervention may improve the speed at which clinicians can respond to pain in this vulnerable population. In this pilot study, we proactively engaged stakeholders (patients, informal caregivers, and providers) in a rural cancer clinic to adapt the Ca-HELP intervention, and its delivery, for use among geriatric cancer patients in this setting.

## Adapting Ca-HELP Intervention

The Cancer Health Empowerment for Living without Pain (Ca-HELP) is an evidence-based communication tool that empowers and engages patients to communicate effectively with their physicians about pain (Kravitz et al., 2009, 2011). The Ca-HELP intervention is rooted in social-cognitive theory (Bandura, 2012; Luszczynska & Schwarzer, 2005), which posits that behavior change and maintenance depend largely on individuals’ ability and self-efficacy to execute a specific behavior. The underlying conceptual model is the Tailored Education and Coaching intervention (TEC**©**) used for Ca-HELP in previous research that found significant improvement among cancer patients in their self-efficacy to communicate about their pain to their oncologists and reductions in pain misconceptions and pain-related impairment (Kravitz et al., 2011). The TEC© tailors messages and skill-building exercises for patients by assessing patient learning goals, values, and needs and by using consumer-friendly coaching messages to improve patients’ self-efficacy to communicate and manage their cancer-related pain in interactions with their doctors (Kravitz et al., 2011).

Ca-HELP coaches patients to ask questions, make requests, and signal distress to their physicians in order to achieve improved pain control. The original intervention workbook consists of six modules: (a) assessment of current knowledge, attitudes, and preferences; (b) clarification and correction of misconceptions about cancer pain control; (c) teaching of relevant concepts (education about cancer pain control); (d) planning (identifying goals of care, creating achievable goals, and creating strategies to communicate these goals of care to providers and family members); (e) rehearsal of communication strategies using role play exercises; and (f) portrayal of learned skills (patient applies skills in visit with health care provider).

Although a promising tool among geriatric cancer patients, Ca-HELP is not currently designed for optimal implementation in rural settings or among older adults. The underlying TEC© conceptual model (Kravitz et al., 2011) primed us to consider how tailoring the Ca-HELP messages and skill building for geriatric cancer patients should include assessment of their learning goals, values, and needs, and incorporate plain-spoken messages based on well-documented rates of low health literacy in rural settings (Halverson et al., 2013). Additionally, adaptation of a candidate evidence-based intervention for use by a target population in a new context can improve its effectiveness and efficiency (Chen et al., 2013; Moore et al., 2021).

The purpose of this article is to describe findings from our interviews with stakeholders that provided the critical insights used to inform adaptations of the Ca-HELP intervention for use in rural settings with geriatric cancer patients. These adaptations took place as a planned formative step in a larger pilot study aimed at testing the preliminary efficacy of the adapted intervention to improve pain self-management in the target population. Guided by the Method for Program Adaptation through Community Engagement (M-PACE; Parker et al., 2012) model which utilizes systematic and detailed feedback from community members and stakeholders to guide adaptation for an appropriate target audience (i.e., geriatric cancer patients in rural settings), we partnered with stakeholders at the pilot study trial site. Key steps of the M-PACE ; Parker et al., 2012) model of adaptation include convening the intended end-users of the intervention (patients, caregivers, and providers) to evaluate the original, unadapted intervention and recommend modifications (Chen et al., 2013). We review findings from semistructured interviews with geriatric cancer patients (*n* = 10), their informal caregivers (*n* = 10), and providers (*n* = 10) working with this population and describe responsive modifications to the Ca-HELP intervention based on their feedback using the Framework for Reporting Adaptations and Modifications-Enhanced model (FRAME; Wiltsey Stirman et al., 2019) for reporting adaptations and modifications to evidence-based interventions. These two models reflect established frameworks for engaging stakeholders in the adaptation process (M-PACE; Parker et al., 2012) and reporting modifications made to the intervention based on user feedback (FRAME; Wiltsey Stirman et al., 2019).

### Reasons to Modify the Original Ca-HELP Intervention

Due to the limited evidence base of interventions targeting pain management communication between older adults with cancer in rural communities and their doctors, the Ca-HELP intervention was selected as a promising candidate intervention that could potentially address this unmet need without expending the time, funding, and other resources needed to develop a new intervention. After reviewing the content and delivery of the original Ca-HELP intervention, the team determined that proactive modifications were needed as a planned and formative step in our pilot study to optimize delivery, content, and implementation in rural settings and among older adults. Guided by the M-PACE (Parker et al., 2012) model, we proactively engaged the intended end users about necessary modifications to improve the fit between the original intervention and a geriatric cancer patient population living in a rural community.

## Research Design and Methods

### Participant Recruitment

Participants were recruited from an outpatient oncology clinic in rural Tennessee. Using a purposive sampling approach (Campbell et al., 2020), a palliative care nurse within the cancer clinic who served as site champion for the research team, used the electronic medical record to identify patients meeting eligibility criteria, including a diagnosis of cancer; being 65 years of age or older; English speaking, residing in a noninstitutional rural setting, receiving care at a community-based rural clinic, able to provide informed consent, and identify an informal caregiver. Informal caregivers, who play a critical role in pain management for cancer patients (Meeker et al., 2011), were eligible if they were 18 years of age or older, identified as providing most of the informal care for the patient, and were able to provide informed consent. Providers were eligible if they were currently working with geriatric cancer patients at the pilot study site. A group of palliative care physicians, oncologists, nurses, and social workers were selected using a purposive sampling technique (Campbell et al., 2020). Consent was obtained verbally by telephone or in-person for all participants. This study, including the process for recruiting participants, received approval from an Institutional Review Board at Maury Regional Medical Center.

### Data Collection

After informed consent was obtained, geriatric cancer patients (*n* = 10), their informal caregivers (*n* = 10), and providers (*n* = 10) were provided with drafts of the original unadapted intervention workbook and asked to review the content. Next, separate one-on-one interviews with patients (*n* = 10) and informal caregivers (*n* = 10) were conducted. Because this phase of the pilot study occurred at the height of the coronavirus diseased 2019 (COVID-19) pandemic, interviews for patients and caregivers occurred via phone as a no-contact method to reduce the risk of COVID-19 virus transmission in light of the vulnerability of this population. Focus groups with providers were conducted using Zoom. Patients and caregivers were compensated $35.00 each for their participation in interviews. Providers were not compensated for their participation in the interviews per health system policy. Sample interview questions are listed in Table 1.

**Table 1.** Sample Interview Questions for Patients, Caregivers, and Providers

Topic	Questions
Overall impressions of Ca-HELP	• Describe the message of Ca-HELP in your own words. • What part of the intervention manual stood out to you most? • What part of the intervention manual was confusing to you? • What words or sentences were difficult to understand? • What part of the intervention manual would be helpful to managing your pain? • What part of the intervention manual would not be helpful to managing your pain? • Overall, how helpful do you think Ca-HELP would be for helping you manage your pain? • What additional thoughts or suggestions do you have for improving Ca-HELP?
Questions about specific components of Ca-HELP	• Do you think the title, “Ca-HELP: Cancer Health Empowerment for Living without Pain” is appropriate? What suggestions do you have to improve the title? • Do you think the content of Ca-HELP was explained in too complicated of terms? If so, which sections? • Does the size of the font or words need to be changed? • Should we change the colors in the Ca-HELP workbook? • How often should the modules be completed? • Where should the modules be completed? (e.g., at home over the telephone, at the doctor’s office) • Do you feel that Ca-HELP relates to your experience in dealing with pain?
Questions about the original six modules of Ca-HELP	• How helpful do you think this module would be for you? • How could we improve this module? • Is there anything that we could add or remove from the module to make it more helpful for older adults with cancer? • How difficult was it for you to understand the content of the module (e.g., language, ideas)? • What made it difficult to understand? (e.g., wording, ideas, amount of information, interactive exercises)? • To what degree was the module helpful in clarifying misconceptions you have about pain management?

*Notes*: Ca-HELP = Cancer Health Empowerment for Living without Pain. Questions for providers were reworded slightly.

### Demographics

Most patients were men (*n* = 8, 80%). Regarding race, six patients were White (60%), three were Black (30%), and one patient did not disclose race (10%). Regarding ethnicity, nine patients self-reported being non-Hispanic/Latino (90%) and one patient did not disclose ethnicity (10%). The mean age of patients was 76.0 years (*SD* = 6.3). Most caregivers were women (*n* = 6, 60%). Regarding race, seven caregivers were White (70%), two were Black (20%), and one caregiver did not disclose race (10%). Regarding ethnicity, eight caregivers self-reported being non-Hispanic/Latino (80%) and two caregivers did not disclose ethnicity (20%). The mean age of caregivers was 64.1 years (*SD* = 13.4), see Table 2.

**Table 2.** Demographics of Stakeholder Patients and Caregivers

Characteristics	Patient		Caregiver	
	Frequency	%	Frequency	%
Total participated	10	100	10	100
Completed demographic survey	10	100		
**Ethnicity**				
Hispanic or Latino	0	0	0	100
Non-Hispanic or Latino	9	90	8	80
Missing	1	10	2	20
**Race**				
White	6	60	7	70
Black	3	30	2	20
Asian or Asian American	0	0	0	0
Pacific Islander	0	0	0	0
Native American	0	0	0	0
Other	0	0	0	0
Missing	1	10	1	10
**Gender**				
Men	8	80	3	30
Women	2	20	6	70
Missing	0	0	1	10

### Methods

Focus groups and interviews with patients, caregivers, and providers were recorded and professionally transcribed. Two health service researchers, a principal investigator and an MPH level research coordinator on the project, cross-checked all transcribed interviews to the original data source and coded interviews separately using a modified grounded theory approach (Strauss and Corbin, 1994). Transcript coding was done by hand with notations and bracketing of themes made directly on the transcripts using an iterative approach of reducing and interpreting initial themes. A process of discussion and peer review of any discrepancies among coders was followed to ensure interrater reliability until consensus was achieved on the analytic categories. Over the course of the analysis, concordant recommendations regarding adaptations to the intervention from patients, caregivers, and providers emerged. Data codes and extracted themes were cross-checked back to the providers through member checking. These recommendations were then categorized according to the FRAME (Wiltsey Stirman et al., 2019) model to systematically track and report how modifications to the original intervention were considered. The research team, which comprised health service researchers and nurse leaders at the pilot study site, collaboratively reviewed the qualitative data for ways to modify the original intervention, including changes and reduction in content, for the targeted intervention group (older adults with cancer in rural settings) in ways that aligned with stakeholder feedback while adhering to the core components of the original intervention. Modifications were tracked and classified according to the FRAME (Wiltsey Stirman et al., 2019) model.

### The Process to Modify the Original Ca-HELP Intervention

Participants were asked about how they might use the original intervention workbooks, the clarity and readability of the workbooks, and the feasibility and utility of the structure of the intervention (e.g., number of sessions and session frequency), see Table 1. Additionally, participants were asked their opinion about necessary modifications in order to make the Ca-HELP intervention workbook content appropriate for the geriatric cancer patient population in a rural community.

Next, we report how interviews with patients, their caregivers, and providers informed modifications to the original Ca-HELP intervention, resulting in a newly adapted version for use among a geriatric cancer population in a rural setting, using elements of FRAME (Wiltsey Stirman et al., 2019).

## Reporting of Modifications to Ca-HELP Using the FRAME Model

The FRAME (Wiltsey Stirman et al., 2019) model provides a systematic approach to develop and report modifications and adaptions to interventions using a series of characteristics, including (a) when and how modifications were made, (b) whether the modification was planned or reactive, (c) who determined the modifications, (d) what is modified, (e) how delivery is impacted, (f) type of modifications to context or content, (g) the impact on fidelity, and (h) the reasons for the modification, including the intent, goal, and context. What follows is a description of stakeholder-informed modifications organized by the framework.

## Results

### A Promising Candidate Intervention

Among the three stakeholder groups, there was no major competing feedback on the recommendations summarized later. Moreover, all stakeholders agreed that the intervention showed potential to address an important problem of pain management of older adults with cancer. For example:

*“The whole idea of looking at myself in the mirror and saying, Okay. I’m a guy. And it’s okay to be honest with pain, about pain. And rather than, Hey, I can just walk it out and I can endure this, and not bother somebody. I think it brought that point home to me, as far as the value of doing that. That’s the main thing I learned from this.” (Patient)*

*“I really wish that anyone with a cancer diagnosis would receive this because they would get better care and pain management overall.” (Caregiver)*

*“What stood out to me is that this would be helpful because my father was very much old school grew up one of eight children, you didn’t ask for anything, and you did not complain about anything, and this tool basically gives permission that you are allowed to have pain and admit that it’s uncomfortable and you need help.” (Caregiver)*

*“I really liked just that it was encouraging patients to advocate for themselves and being specific with their physician about where their pain is and how often and those types of just encouraging patients to take that active role in talking with their physician. I really liked that.” (Provider)*

Next, we report on the nature of modifications made to the delivery, context, and content of the intervention made during the preimplementation phase with illustrative quotes from stakeholders about the rationale for these changes. Sample modifications are summarized in Table 3.

**Table 3.** Sample Modifications to Ca-HELP Resulting From Patient, Caregiver, and Provider Input

Participant type	FRAME© characteristic	Illustrative quote	Modifications
Provider	Context	*“The cancer clinic is extremely busy during normal times. With COVID, we are busier than ever and try to limit how often some of our older patients come into the clinic to reduce exposing them to anything since their immune systems are weak.”*	Reduced delivery from several sessions to a single session with nurse delivered via telephone
Patient (older adult with cancer)	Content	*“I’m not sure what Ca-HELP means on the front cover.”*	Retitled patient workbook from “Ca-HELP For Pain Management among Older Adults” to “5 Steps for Talking with your Doctor about Pain when Living with Cancer”
Provider	Content	*“I think when people flip through it and say, you know, so many pages of all of these things, I have to quote unquote, read, then that’s what sort of turns them off. Replacing some text with more graphics may be helpful.”*	Reduced number of pages in patient workbook from 24 to 18, used simple image-based communication
Caregiver	Content	*“They might need to go up a font size because if you’re dealing with elderly patients, or you could make a separate one that’s got the bigger, larger type style for the elderly, that would help because sometimes some of these things, they kind of run together.”*	Increased font size to accommodate low levels of health literacy and vision challenges in target population of older adults with cancer
Patient (older adult with cancer)	Content	*“Make the language simpler and easier to understand, more lay language. The material was confusing. I didn’t know what was what.”*	Simplified text by replacing jargon, changed formal terms, like “module” to informal terms like “step”
Caregiver	Content	*“Rehearsing and applying modules could be combined.”*	Combined original modules 5 and 6 (rehearsing and role-playing communication strategies for patients to talk about pain with doctor) into a combined “step”

*Notes*: Ca-HELP = Cancer Health Empowerment for Living without Pain; FRAME = Framework for Reporting Adaptations and Modifications-Enhanced model.

### Modifications to Original Intervention Delivery

Informed by stakeholder feedback, the project team made contextual modifications to the format of the intervention. The original intervention, in which the patient completes the intervention workbook about how to talk with their doctor about pain, was delivered across several sessions between a patient and a health coach. However, the context of delivering the intervention in a busy community cancer clinic during the COVID-19 pandemic necessitated changes to the delivery. For example:

*“The cancer clinic is extremely busy during normal times. With COVID, we are busier than ever and try to limit how often some of our older patients come into the clinic to reduce exposing them to anything since their immune systems are weak.”* (Provider)

In response to the context of COVID-19, we reduced the intervention delivery from the original format of several sessions with a health coach to a single telephone session delivered by a palliative care nurse working in the cancer clinic.

*“I think we’ve got a mixed patient population where we have some that are just not very health literate. And in fact, maybe not very literate, you know, in some respects and so if someone’s assisting them with this, I can see that it would get completed.” (Provider)*

In addition to documenting these changes in the intervention training manual used to standardize implementation, the project team discussed these and all modifications to ensure they were fidelity-consistent. These discussions were informed by a formative meeting with the intervention developer (Kravitz et al., 2009, 2011) about the function of the original intervention’s core elements.

### Modifications to Original Intervention Context

Across the three stakeholder groups (patients, caregivers, and providers), there was unanimous feedback that the Ca-HELP tool should be shortened from its original format as a 24-page patient-facing workbook with six communication modules, for example:

“The biggest problem I have is getting him to look at it to read it.” (Caregiver)

*“I think when people flip through it and say, you know, so many pages of all of these things, I have to quote unquote, read, then that’s what sort of turns them off. Replacing some text with more graphics may be helpful.” (Provider)*

As such, we condensed the number of words on each page, reducing the total number of pages from 24 to 18 with only 8 “active” or “workbook” pages. Additional contextual changes were made to accommodate low levels of health literacy and vision challenges in the target population. Changes included reformatting the patient-facing booklet to reduce the number of words on each page and increasing the font-size for text.

*“They might need to go up a font size because if you’re dealing with elderly patients, or you could make a separate one that’s got the bigger, larger type style for the elderly, that would help because sometimes some of these things, they kind of run together.” (Caregiver)*

In addition to the reduction of text, stakeholders recommended adding more pictures to communicate the intervention elements. This was in response to health literacy concerns, as well as, addressing patient’s the resistance to discussing pain.

*“I would limit the number of texts, bigger lettering, and just be clearer, okay, this is to help you understand pain or learn discuss pain with your doctor, period, you know, just simplifying it and, yeah, there’s just too many things to write and to answer about, maybe keeping it simple.” (Provider)*

To enhance relatability and engagement with the workbook, pain scales and descriptions of pain were changed from word-based intakes to engaging and colorful visuals. For example, in Step 1, the nurse uses the workbook to guide the patient through a series of questions about the type of pain they are having. In the original intervention, the question, “If you have pain, what does your pain feel like?” had text-only responses. In the adapted intervention, we modified the responses to this question to include a visual associated with each word, such as a fire symbol with the response of burning pain.

### Modifications to Original Intervention Content

All stakeholders unanimously recommended modifications to simplify the intervention content for use among geriatric patients in a rural setting. For example:

“I’m not sure what Ca-HELP means on the front cover.” (Patient)

*“Make the language simpler and easier to understand, more lay language. The material was confusing. I didn’t know what was what.” (Patient)*

“Rehearsing and applying modules could be combined.” (Caregiver)

*“Speaking up about pain (Module 3) is relevant and important, but distill it down to a few key sentences.” (Provider)*

Modifications to simplify the tool included removing jargon and using more consumer-friendly language. To begin, titles were reworded to be patient-centered, rather than researcher-centered. We retitled the original front cover of the patient workbook from “Ca-HELP For Pain Management among Older Adults” to “5 Steps for Talking with your Doctor about Pain when Living with Cancer.” Additionally, stakeholder feedback drew our attention to how each module in the original intervention was titled in a way that referred to the goal of the module from the perspective of researchers, rather than the intended end users, patients. For example, in the original intervention, “Module 3: Knowledge Transferring (TEACH) was modified to “Step 3: Learn How to Self-Manage and Talk with Your Doctor About Your Pain.” Additional jargon was modified in response to stakeholder request for simple, lay-person language. The original booklet contained six modules in the patient-facing workbook. We replaced the word “module” with the word “step.” We also reduced the number of steps from six to five by combining the final two steps in the intervention into one (rehearsal of communication strategies using role play exercises and portrayal of learned skills when the patient applies skills in a visit with health care provider).

## Limitations

This study has several limitations. First, limited demographics included a predominately White, non-Hispanic/Latino sample. However, this was reflective of the patient population in rural Tennessee. Second, study participation required the inclusion of an informal caregiver. Patients who lack informal caregivers may need a pain management communication tool tailored to their unique needs. Third, patients were not screened for pain as part of the eligibility criteria to participate in the stakeholder group to inform adaptations. The rationale for this was that there could be stigma associated with older patients with cancer admitting pain, reporting pain, and seeking relief. (Cleeland, 1998; Hachem et al., 2019; Reyes-Gibby et al., 2006) Fourth, the eligibility criteria for patient participants included a diagnosis of cancer but did not require a specific disease type or severity/stage. This formative aim in our pilot study, targeting older adults with cancer, was part of a multiphase pragmatic trial embedded in a busy rural cancer clinic during the COVID-19 pandemic. To make the study as pragmatic as possible for nursing staff and our recruitment needs, we used broad inclusion criteria for cancer. Fifth, as noted in the FRAME (Wiltsey Stirman et al., 2019) model, the degree to which some modifications, such as reducing the intervention from several sessions to a single session, affect fidelity is not fully known. The intent of the modifications was to respond to the context of COVID-19, maximize fit, and ensure implementation success. Finally, not all invited local stakeholders participated in our project. It is possible that the perspective of stakeholders who did not participate are different from those who were interviewed which could result in different modifications to the original intervention.

## Discussion and Implications

Our findings suggest that older adults living with cancer, their informal caregivers, and providers agree that pain management in rural settings is a problem. Due to a lack of evidence-based interventions to specifically address pain for this target population and setting, we selected a promising candidate intervention, Ca-HELP, based on previous research indicating significant improvement among cancer patients in their self-efficacy to communicate with their doctors about their pain (Kravitz et al., 2009, 2011). Guided by the prior work of Chen et al. and the M-PACE model (Chen et al., 2013; Parker et al., 2012), we engaged stakeholder groups whom the Ca-HELP intervention is intended to target (i.e., older adults, informal caregivers, and providers in rural settings) to proactively solicit reactions and suggested modifications to optimize fit (Chen et al., 2013; Parker et al., 2012).

All stakeholders agreed that the original, unadapted Ca-HELP intervention, which coaches patients to ask questions, make requests, and signal distress to their physicians in order to achieve improved pain control showed potential to address this problem. Patients, informal caregivers, and providers suggested that the original intervention delivery, context, and content be modified for the new target population, older adults living with cancer in rural settings.

A multidisciplinary team of nurse leaders and researchers evaluated stakeholder feedback and recommendations before determining which adaptations were made. Adaptations were cataloged and reported using the FRAME (Wiltsey Stirman et al., 2019) model. Modifications included reducing the delivery to a single telephone session offered by a nurse prior to meeting with the provider, retitling the cover page to be more patient-centered, reducing the number of pages in the intervention workbook, reducing the number of words, eliminating jargon, using larger font, combining modules, and increasing the use of pictures to communicate complex health information and descriptions of pain. The next step in this multiphase pilot study is to evaluate the feasibility and acceptability of the adapted Ca-HELP intervention among older adults with cancer in rural clinic settings and test the preliminary efficacy of the Ca-HELP intervention adaptation on older adults with cancer to improve pain self-management, pain misconceptions, and self-efficacy to communicate with their physicians regarding pain severity.

Given the lack of evidence-based interventions targeting pain management for older adults in rural settings, identifying promising interventions and engaging stakeholders in the process to modify them can improve the speed, efficiency, and fit of interventions to address this unmet need. Pilot studies represent a unique opportunity for this type of early-stage work through the support of stakeholder engagement to inform intervention adaptation. This critical groundwork requires engaging the intended end users of interventions to make planned modifications to tailor programs for target populations and contexts as formative steps (Chen et al., 2013; Parker et al., 2012). In our pilot study, we engaged older adults living with cancer, their informal caregivers, and providers in rural settings for their feedback. Engaging older adults living with cancer in rural communities in the adaptation of a promising evidence-based intervention is a patient-centered research approach that improves the fit of the intervention and empowers rural older adult patients to communicate effectively with their physicians about pain. It also addresses a lack of relevancy between research and stakeholders’ everyday lives, which is an avoidable problem that affects the adoption and implementation of critical research interventions in the real-world (Harrison et al., 2020). A major benefit of the stakeholder input was optimizing fit of the intervention of Ca-HELP for rural older adults, although these improvements have the potential to improve uptake and success of Ca-HELP for all cancer patients.

Older adults with cancer in rural settings have undertreated pain, are more likely to hide pain symptoms, and avoid expressing the need for pain relief (Beck et al., 2009; Cleeland, 1998; Dalton et al., 1995; Hachem et al., 2019; Reyes-Gibby et al., 2006). There is an urgent need for interventions that can be implemented effectively to help older adults living with cancer in these vulnerable communities communicate with doctors about their pain. Despite evidence that the Ca-HELP improves the self-efficacy of patients to talk to their doctor about their pain and reduces pain misconceptions, only a limited number of studies have implemented this promising intervention. Modifying a candidate intervention is innovative and a social enterprise that involves planning and partnership between researchers and stakeholders. Our multistakeholder team proactively and collaboratively made context and content modifications to the Ca-HELP intervention tool using feedback from patients, informal caregivers, and providers with a goal to optimize fit without compromising the active ingredients. Documenting and reporting modifications to interventions are critical to their implementation and will lay the groundwork for further testing of the efficacy of the adapted Ca-HELP intervention.
